# Empirical comparison of item response theory models with rater's parameters

**DOI:** 10.1016/j.heliyon.2018.e00622

**Published:** 2018-05-08

**Authors:** Masaki Uto, Maomi Ueno

**Affiliations:** The University of Electro-Communications, 1-5-1 Chofugaoka, Chofu, Tokyo 182-8585, Japan

**Keywords:** Psychology, Information science

## Abstract

In various assessment contexts including entrance examinations, educational assessments, and personnel appraisal, performance assessment by raters has attracted much attention to measure higher order abilities of examinees. However, a persistent difficulty is that the ability measurement accuracy depends strongly on rater and task characteristics. To resolve this shortcoming, various item response theory (IRT) models that incorporate rater and task characteristic parameters have been proposed. However, because various models with different rater and task parameters exist, it is difficult to understand each model's features. Therefore, this study presents empirical comparisons of IRT models. Specifically, after reviewing and summarizing features of existing models, we compare their performance through simulation and actual data experiments.

## 1 Introduction

The need to measure practical and higher order abilities such as problem solving, critical reasoning, and creative thinking skills has recently increased in various assessment contexts (Bernardin et al., 2016; Kassim, 2011; Muraki et al., 2000; Myford and Wolfe, 2003; Uto and Ueno, 2016). To measure such abilities, performance assessment by raters, which evaluates examinees' outcomes or processes for performance tasks, has attracted much attention (Muraki et al., 2000; Palm, 2008; Wren, 2009). Performance assessment has been used in various formats such as essay writing tests, speaking tests, interview examinations, and group discussion tests.

However, difficulty persists that the ability measurement accuracy depends strongly on rater and task characteristics (Bernardin et al., 2016; Eckes, 2005; Kassim, 2011; Myford and Wolfe, 2003, Myford and Wolfe, 2004; Nguyen et al., 2015; Saal et al., 1980; Shah et al., 2014; Suen, 2014). Some rater and task characteristics on which the accuracy generally depends are rater severity, consistency, range restriction, task difficulty, and discrimination. Therefore, improving measurement accuracy requires ability estimation considering effects of those characteristics (Muraki et al., 2000; Suen, 2014; Uto and Ueno, 2016).

For this reason, many item response theory (IRT) models that incorporate rater and task characteristic parameters have been proposed (Linacre, 1989; Patz and Junker, 1999; Patz et al., 1999; Ueno and Okamoto, 2008; Uto and Ueno, 2016). These models can estimate the abilities of examinees considering these characteristics. Therefore, they are known to provide more accurate ability measurement than average or total scores do (Eckes, 2015; Ueno and Okamoto, 2008; Uto and Ueno, 2016). However, understanding the features and performance of each model is difficult because existing models incorporate different rater and task characteristic parameters. Although many applications use a specific model to measure examinee ability or to analyze rater and task characteristics from actual performance assessment data (e.g., Eckes, 2005, Eckes, 2015; Kassim, 2011; Myford and Wolfe, 2004; Patz and Junker, 1999; Patz et al., 1999; Rahman et al., 2017; Ueno and Okamoto, 2008), no report of the relevant literature describes a study that has compared the features and performance of existing models.

For that reason, this study presents empirical comparisons of IRT models that incorporate rater and task parameters. Specifically, we first review and summarize the features of existing models. Then we compare their performance through simulation and actual data experiments. To clarify the features and performance of those models, the comparisons are conducted while changing the following conditions: 1) the numbers of examinees, tasks, and raters, 2) the characteristics of raters and tasks (specifically, rater severity, consistency, range restriction, task difficulty, and discrimination).

It is noteworthy that Uto and Ueno (2016) conducted a model comparison to demonstrate the effectiveness of their proposed model, assuming peer assessment situations in which examinees do mutual assessment. The study demonstrated that their model provides higher ability measurement accuracy than the other models when raters and examinees become numerous. However, in general performance assessment situations, the raters are far fewer than the examinees. The study did not evaluate the models in such situations. Additionally, the study ignored the effects of task quantity, and ignored how each rater and task characteristic affect model performance. Our study compared features and performance of existing models considering the effects of various rater and task characteristics with changing assessment settings, such as the number of raters, examinees and tasks. Therefore, our study is sufficiently different from earlier ones by Uto and Ueno (2016). The results of our study are expected to be helpful in elucidating features of existing models and in choosing a model that provides better performance in an actual assessment situation.

## 2 Design

### 2.1 Performance assessment data

We assume that performance assessment data ***U*** consist of a rating $x_{ijr}$ given by rater r∈R={1,…,R} to an outcome of examinee j∈J={1,…,J} for performance task i∈I={1,…,I}. That is, the data ***U*** are defined as equation (1).

(1) $$U=\{x_{ijr}|i\in I,j\in J,r\in R\}.$$

If a rating has been given, then $x_{ijr}=k$ for some rating category k∈{1,…,K} and $x_{ijr}=-1$ represents missing data. Consequently, $x_{ijr}\in K=\{-1,1,\ldots ,K\}$.

The aim of this study is to measure the ability of examinees accurately from the rating data.

### 2.2 Task and rater biases in performance assessment

As described in Section 1, ability measurement accuracy is known to depend on rater and task characteristics (Bernardin et al., 2016; Eckes, 2005; Kassim, 2011; Myford and Wolfe, 2003, Myford and Wolfe, 2004; Nguyen et al., 2015; Saal et al., 1980; Shah et al., 2014; Suen, 2014).

Common rater characteristics on which the accuracy generally depends are the following:

1. *Severity*: The tendency to give consistently lower ratings than are justified by the outcomes (Kassim, 2011).
2. *Consistency*: The extent to which the rater assigns similar ratings to outcomes of similar quality (Kassim, 2011).
3. *Restriction of range*: The tendency to overuse a few restricted rating categories (Kassim, 2011; Myford and Wolfe, 2003; Saal et al., 1980).

Furthermore, typical task characteristics on which the accuracy depends are presented below.

1. *Difficulty*: More difficult tasks tend to engender consistently lower ratings.
2. *Discrimination*: The extent to which different levels of the ability to be measured are reflected in the quality of outcomes in the task.

To measure examinees' ability reflecting these rater and task characteristics, many item response theory (IRT) (Lord, 1980) models that incorporate parameters representing those characteristics have been proposed. Before reviewing the models, the following section describes the traditional IRT models that are the fundamental basis for those IRT models.

## 3 Theory

### 3.1 Item response theory

IRT, a test theory based on probabilistic models, defines the response probability of an examinee to a test item as a function of the latent ability of the examinee and item characteristics. IRT enables estimation of examinee ability considering characteristics of test items (e.g., difficulty and discrimination). Therefore, IRT generally realizes more accurate ability measurement than average or total scores do. Another advantage of IRT is that the abilities of examinees who took different test items can be estimated on the same scale. Based on those advantages, IRT has been used in various testing situations (e.g., Carlson and von Davier, 2013; de Ayala, 2009; Information Technology Promotion Agency, 2017; Reise and Revicki, 2014).

The following subsections describe the two IRT models used as basis models in this study: the Graded Response Model (GRM) (Samejima, 1969) and the Generalized Partial Credit Model (GPCM) (Muraki, 1997).

### 3.2 Graded response model

The GRM gives the probability that examinee *j* obtains category *k* in item *i* as equations (2) and (3).

(2) $$P_{ijk}=P_{ij(k-1)}^{⁎}-P_{ijk}^{⁎},$$

(3) $$\text{where }\left\{ \begin{array}{cc} P_{ij0}^{⁎}=1\\ P_{ijk}^{⁎}={\left[1+\exp(-\alpha_{i}(\theta_{j}-b_{ik}))\right]}^{-1}, & 1<k<K-1\\ P_{ijK}^{⁎}=0. \end{array}\right.$$

In those equations, $\theta_{j}$ represents the ability of examinee *j*, $\alpha_{i}$ is the discrimination parameter of item *i*, and $b_{ik}$ is a difficulty parameter that denotes the upper grade threshold parameter for category *k* of item *i*. Here, the order of the difficulty parameters is $b_{i1}<b_{i2}<\cdots <b_{i(K-1)}$.

### 3.3 Generalized partial credit model

The GPCM gives the probability $P_{ijk}$ as equation (4).

(4) $$P_{ijk}=\frac{\exp\sum_{m=1}^{k}\left[\alpha_{i}(\theta_{j}-\beta_{im})\right]}{\sum_{l=1}^{K}\exp\sum_{m=1}^{l}\left[\alpha_{i}(\theta_{j}-\beta_{im})\right],}$$

where $\beta_{ik}$ is a step difficulty parameter that denotes the difficulty of transition between category k−1 and category *k* for item *i*. Here, the problem of model non-identifiability arises in this model. In a non-identifiable model, values of the parameters cannot be uniquely determined because different sets of the values provide the same response probability (San Martín et al., 2015; van der Linden, 2016a). The non-identifiability is generally eliminated by fixing some parameter values or by fixing a mean over a parameter set (e.g., Muraki, 1992; Uto and Ueno, 2016; van der Linden, 2016a). In this model, $\beta_{i1}=0$ for each *i* is given for model identification.

The GPCM is often described by decomposing the step difficulty parameter $\beta_{ik}$ into $\beta_{i}+d_{ik}$ as equation (5).

(5) $$P_{ijk}=\frac{\exp\sum_{m=1}^{k}\left[\alpha_{i}(\theta_{j}-\beta_{i}-d_{im})\right]}{\sum_{l=1}^{K}\exp\sum_{m=1}^{l}\left[\alpha_{i}(\theta_{j}-\beta_{i}-d_{im})\right],}$$

where $\beta_{i}$ is a positional parameter reflecting the overall difficulty of item *i* and $d_{ik}$ is a threshold parameter denoting the difficulty of transition between category k−1 and category *k* for item *i*. Here, $d_{i1}=0$ and $\sum_{k=2}^{K}d_{ik}=0$ for each *i* are given for model identification.

The GPCM has many sub-models. Specifically, the partial credit model (PCM) (Masters, 1982) is a special case of GPCM when $\alpha_{i}=1.0$ for all items. The rating scale model (RSM) (Andrich, 1978) is a special case of the PCM when $\beta_{ik}$ is decomposed to $\beta_{i}+d_{k}$. Here, $d_{k}$ is a category parameter representing the difficulty of transition between category k−1 and category *k*.

### 3.4 Interpretation of item parameters in polytomous IRT models

This subsection presents a detailed explanation of the item characteristic parameters incorporated in the polytomous IRT models. The following explanations are based on the equation (5) form of the GPCM, which has the most numerous item parameters of all the models described above.

**Figure 1:**
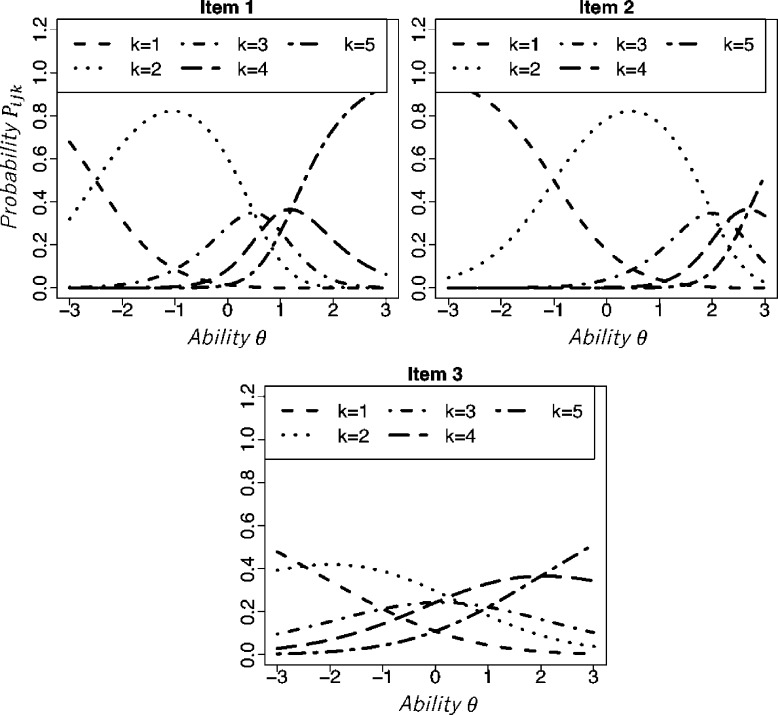
Item response curves of the generalized partial credit model for five categories.

Figure 1 depicts the item response curves (IRCs) of the GPCM for three items with different item parameters. Here, we used parameters $\alpha_{i}=1.5$, $\beta_{i}=0.0$, $d_{i2}=-2.5$, $d_{i3}=0.5$, $d_{i4}=0.8$, and $d_{i5}=1.2$ for *Item 1* (upper-left panel); $\alpha_{i}=1.5$, $\beta_{i}=1.5$, $d_{i2}=-2.5$, $d_{i3}=0.5$, $d_{i4}=0.8$, and $d_{i5}=1.2$ for *Item 2* (upper-right panel); and $\alpha_{i}=0.5$, $\beta_{i}=0.0$, $d_{i2}=-2.5$, $d_{i3}=0.5$, $d_{i4}=0.0$, and $d_{i5}=2.0$ for *Item 3* (lower panel). The horizontal axis shows the latent ability *θ*. The vertical axis shows probability $P_{ijk}$.

Figure 1 shows that examinees with lower (higher) ability tend to obtain scores in lower (higher) categories.

The difficulty parameter $\beta_{i}$ controls the location of the IRC. As the value of this parameter increases, the IRC shifts to the right. One can compare the IRCs for *Item 2* with those for *Item 1*. It denotes that obtaining higher categories is more difficult in items with higher difficulty parameter values.

The item discrimination parameter $\alpha_{i}$ controls differences in response probabilities among the categories. The lower the item discrimination is, the smaller the difference is, as shown by the IRCs for *Item 3* in Figure 1. Those trends imply that, in a lower discrimination item, the randomness of categories given to a specific examinee is increased. Low discrimination items generally engender low ability measurement accuracy because the observed data do not necessarily correlate with true ability.

Parameter $d_{ik}$ represents the location on the *θ* scale at which the adjacent categories, *k* and k−1, are equally likely to be observed (Eckes, 2015; Sung and Kang, 2006). Therefore, when the difference of $d_{i(k+1)}-d_{ik}$ increase, the probability of obtaining category *k* increases over widely various ability scales. In Figure 1, $d_{i3}-d_{i2}$ is large for *Item 1* and *Item 2*. Therefore, the response probability for category 2 had a high value.

### 3.5 Assumption of IRT

IRT generally requires two major assumptions: Unidimensionality and local independence (e.g., Nering and Ostini, 2010; Reise and Revicki, 2014; van der Linden, 2016a). The assumption of unidimensionality is that one latent ability is measured in a test. The local independence assumption implies responses given to different items are mutually independent given the ability. Therefore, the joint probability of responses to multiple items is equal to the product of the response probability to each item conditioning on the ability.

Another assumption of IRT is that all bias factors affecting item responses are incorporated into the model. This assumption is necessary to represent the response probability for given data precisely (de Ayala, 2009). However, the increase of the parameter number requires more data to estimate the parameters and ability accurately (e.g., Reise and Revicki, 2014; Uto and Ueno, 2016; Waller, 1981). Therefore, we should practically select a model that represents bias factors as precisely as possible using the fewest parameters.

## 4 Model

### 4.1 IRT models that incorporate rater parameters

The IRT models introduced above are applied to two-way data that consist of examinees and test items. However, as described in Subsection 2.1, performance assessment data are three-way data consisting of examinees, tasks, and raters. Therefore, they are not directly applicable to performance assessment. To resolve that difficulty, many IRT models that incorporate rater characteristic parameters have been proposed (e.g., Linacre, 1989; Patz and Junker, 1999; Patz et al., 1999; Ueno and Okamoto, 2008; Uto and Ueno, 2016). In the models, the item characteristic parameters are regarded as task characteristic parameters. The following subsections describe these models. It is noteworthy that the following IRT models also assume unidimensionality and local independence, as explained in the previous subsection (Eckes, 2015; Esfandiari et al., 2013; Ilhan, 2016).

### 4.2 Many-faceted Rasch model

The many-faceted Rasch model (MFRM) (Linacre, 1989) is a traditional IRT model that incorporates rater and task parameters. Although several MFRM variations are known to exist (Eckes, 2015; Myford and Wolfe, 2003, Myford and Wolfe, 2004), the most common formation is defined as a PCM that incorporates a rater severity parameter. The MFRM provides the probability that rater *r* responds with category *k* to examinee *j*'s outcome for task *i* as equation (6).

(6) $$P_{ijrk}=\frac{\exp\sum_{m=1}^{k}\left[\theta_{j}-\beta_{i}-\beta_{r}-d_{m}\right]}{\sum_{l=1}^{K}\exp\sum_{m=1}^{l}\left[\theta_{j}-\beta_{i}-\beta_{r}-d_{m}\right],}$$

where positional parameter $\beta_{i}$ denotes the difficulty of task *i*, positional parameter $\beta_{r}$ denotes the severity of rater *r*, and $d_{k}$ is a category parameter that represents the difficulty of transition between categories k−1 and *k*. Here, $\beta_{r=1}=0$, $d_{1}=0$ and $\sum_{k=2}^{K}d_{k}=0$ are given for model identification.

A unique MFRM feature is that it is defined by the fewest parameters in existing IRT models with task and rater parameters. The accuracy of parameter estimation generally increases as the number of parameters per datum decreases (Bishop, 2006; Reise and Revicki, 2014; Uto and Ueno, 2016; Waller, 1981). Consequently, MFRM can estimate model parameters from a small dataset more accurately than the other models can.

By contrast, the MFRM relies on the assumption that all tasks have the same discriminatory power, although this assumption is not practically satisfied (DeCarlo, 2005; Patz and Junker, 1999; Patz et al., 1999; Ueno and Okamoto, 2008; Uto and Ueno, 2016). To relax this constraint, extensions of GPCM and GRM, which allow the discrimination power to differ among tasks, have been proposed.

### 4.3 GPCM and GRM extensions that incorporate rater parameters

One model proposed by Patz and Junker (1999) is a GPCM that incorporates a rater severity parameter. The model provides response probabilities $P_{ijrk}$ as equation (7).

(7) $$P_{ijrk}=\frac{\exp\sum_{m=1}^{k}\left[\alpha_{i}(\theta_{j}-\beta_{im}-\rho_{ir})\right]}{\sum_{l=1}^{K}\exp\sum_{m=1}^{l}\left[\alpha_{i}(\theta_{j}-\beta_{im}-\rho_{ir})\right],}$$

where $\alpha_{i}$ is a discrimination parameter for task *i*, $\beta_{ik}$ is a step difficulty parameter that denotes the difficulty of transition between categories k−1 and *k* in task *i*, and $\rho_{ir}$ reflects the severity of rater *r* for task *i*. Here, $\beta_{i1}=0$ and $\rho_{i0}=0$ are given for model identification. A unique feature of this model is the incorporation of a different rater severity for each task. When the severity of raters is likely to change between tasks, the model will fit the data well.

Ueno and Okamoto (2008) proposed a GRM that incorporates rater severity parameters. In this model, the response probabilities are given as equations (8) and (9).

(8) $$P_{ijrk}=P_{ijr(k-1)}^{⁎}-P_{ijrk}^{⁎},$$

(9) $$\text{where }\left\{ \begin{array}{c} P_{ijr0}^{⁎}=1,\\ P_{ijrk}^{⁎}={\left[1+\exp(-\alpha_{i}(\theta_{j}-b_{i}-\varepsilon_{rk}))\right]}^{-1},1<k<K-1\\ P_{ijrK}^{⁎}=0. \end{array}\right.$$

In those expressions, $b_{i}$ represents the difficulty of task *i*, $\varepsilon_{rk}$ denotes the difficulty in obtaining category *k* for rater *r*. Here, $\varepsilon_{r1}<\varepsilon_{r2}<\cdots <\varepsilon_{rK-1}$. Additionally, $\varepsilon_{11}=-1.0$ is given for model identification. The model has the unique feature that it can represent the range restriction characteristics of raters. The characteristics can be represented by $\varepsilon_{rk}$, as explained in Subsection 5.1.

Uto and Ueno (2016) proposed another GRM that incorporates rater parameters. In this model, the response probabilities are given as equations (10) and (11).

(10) $$P_{ijrk}=P_{ijrk-1}^{⁎}-P_{ijrk}^{⁎},$$

(11) $$\text{where }\left\{ \begin{array}{c} P_{ijr0}^{⁎}=1,\\ P_{ijrk}^{⁎}={\left[1+\exp(-\alpha_{i}\alpha_{r}(\theta_{j}-b_{ik}-\varepsilon_{r}))\right]}^{-1},1<k<K-1\\ P_{ijrK}^{⁎}=0. \end{array}\right.$$

In those equations, $\alpha_{r}$ reflects the consistency of rater *r*, $\varepsilon_{r}$ represents the severity of rater *r*, and $b_{ik}$ denotes the difficulty in obtaining category *k* for task *i* (with $b_{i1}<b_{i2}<\cdots <b_{iK-1}$). Here, $\alpha_{r=1}=1$ and $\varepsilon_{1}=0$ are assumed for model identification. The model has two features: 1) it incorporates a rater consistency parameter; and 2) the parameters are the second fewest when the number of raters is large. Therefore, the model is expected to be suitable when the rater consistency varies and when the raters become numerous.

### 4.4 Hierarchical rater model

The models above are defined as IRT models incorporating the rater characteristic parameters directly. As another modeling approach, hierarchical rater models (HRM) have been proposed (DeCarlo et al., 2011; Lu and Wang, 2006; Patz et al., 1999). HRMs assume the existence of a latent ideal rating $\xi_{ij}$ for each outcome. Furthermore, they define the rating process as a two-stage process. Concretely, a HRM proposed by Patz et al. (1999) hierarchy connects two rating processes using an IRT model and a signal detection model. In the first stage, examinee *j*'s outcome for task *i* has ideal rating $\xi_{ij}$ is to be obtained from the following PCM.

(12) $$p(\xi_{ij}=k|\theta_{j},\beta_{i},d_{i})=\frac{\exp\sum_{m=1}^{k}\left[\theta_{j}-\beta_{i}-d_{im}\right]}{{\sum_{l=1}}^{K}\exp\sum_{m=1}^{l}\left[\theta_{j}-\beta_{i}-d_{im}\right]}$$

Here, $d_{i1}=0$ and $\sum_{k=2}^{K}d_{ik}=0$ for each *i* are assumed for model identification.

Then, in the second stage, rater *r*'s response $x_{ijr}$ to examinee *j*'s outcome for task *i* is assumed to be obtained from the following signal detection model (Peterson et al., 1954) given the ideal rating $\xi_{ij}$ as equation (13).

(13) $$p(x_{ijr}=k|\xi_{ij})\propto exp\left\{\frac{-{\left[k-(\xi_{ij}+\sigma_{r})\right]}^{2}}{2\psi_{r}^{2}}\right\},$$

where $\sigma_{r}$ denotes a rater's severity and the reciprocal of $\psi_{r}^{2}$ denotes a rater's consistency.

A unique feature of the HRM is its incorporation of an ideal rating for each outcome. Another feature is the incorporation of the rater consistency parameter, which has been used only in Uto and Ueno (2016).

### 4.5 Other statistical models

Several statistical models that are applicable to performance assessment data without IRT models have also been proposed (e.g., Goldin, 2012; Piech et al., 2013). However, those models cannot estimate examinee ability because they have no variable representing ability. Therefore, we are not concerned with these non-IRT-based models.

## 5 Analysis

As described above, IRT models with various rater and task characteristic parameters have been proposed. However, no relevant studies have clarified their features and performance, as explained in Section 1.

For that reason, we present empirical comparisons of the IRT models. First, the following subsections present summaries of IRT model features. Then we compare their performance through simulation experiments. Hereinafter, we designate the models of (6) as *MFRM*, (7) as *Patz1999*, (8) as *Ueno2008*, (10) as *Uto2016*, and (12) and (13) as *HRM*.

### 5.1 Comparison of task and rater characteristics assumed in each model

In this section, we explain the rater and task characteristics considered in the IRT models. Table 1 summarizes the characteristics presented in each model.

**Table 1:** Task and rater characteristics in each model, and the number of parameters.

Model	Task characteristics	Rater characteristics	Number of parameters
MFRM	Difficulty	Severity	*I* + *K* + *R* + *J* − 2

Patz1999	Discrimination	Severity for each task	*I*(*K* + *R* − 1)+*J*
	Difficulty for each category		

Ueno2008	Discrimination	Severity	2*I* + *R*(*K* − 1)−1 + *J*
	Difficulty	Range restriction	

Uto2016	Discrimination	Severity	*IK* + 2(*R* − 1)+*J*
	Difficulty for each category	Consistency	

HRM	Difficulty for each category	Severity	*I*(*K* − 1 + *J*)+2*R* + *J*
		Consistency	

Table 1 shows that all the models can reflect task difficulty and rater severity. However, as described in Section 4, each model has the following unique features:

1. MFRM is the simplest model that incorporates only task difficulty and rater severity parameters.
2. Patz1999 allows the rater's severity to differ among tasks.
3. Ueno2008 is the only model that can consider the range restriction characteristic of raters. Ueno2008 relies on the assumption, however, that the difficulty of obtaining each category is the same over all the tasks, although Patz1999, Uto2016, and HRM allow them to be different.
4. Uto2016 and HRM can reflect differences in rater consistency.

To explain how the rater characteristics are represented by each model parameter, the IRCs of Patz1999, Ueno2008, and Uto2016 for raters with different characteristics are presented in Figure 3.

**Figure 2:**
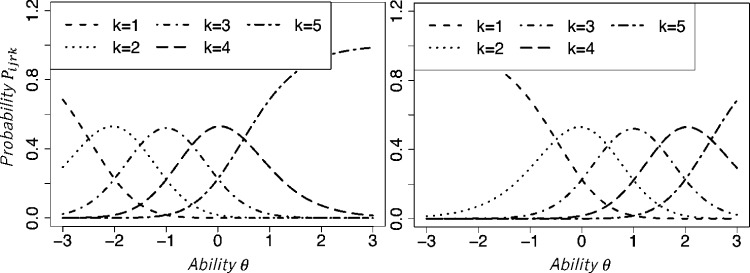
Item response curves of Patz1999 for two raters with different rating severity.

As described before, all models represent rater severity. Specifically, it is represented by $\beta_{r}$ in MFRM, $\rho_{ir}$ in Patz1999, $d_{rk}$ in Ueno2008, $\epsilon_{r}$ in Uto2016, and $\sigma_{r}$ in HRM. As the parameter values increases, the IRC shifts to the right, which indicates that raters tend to assign low scores consistently. This point is presented in Figure 2 for the Patz1999 model. Here, we used the lower severity value $\rho_{ir}=-1.0$ for the left panel and the higher value $\rho_{ir}=1.0$ for the right panel. Other model parameters were the same. Figure 2 shows that the IRC of the severe rater is further right than that of the lenient rater. Furthermore, Patz1999 allows a change of rater severity among tasks, although the other models incorporate the assumption that the rater severity is constant among tasks.

**Figure 3:**
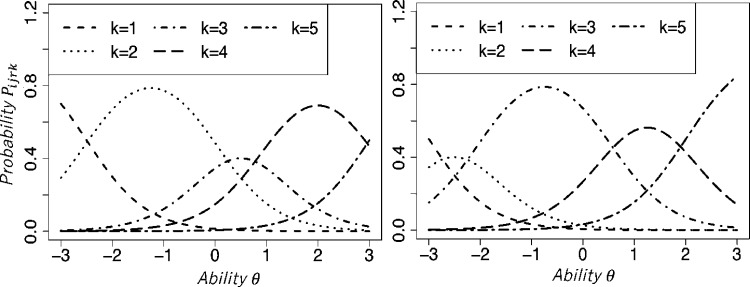
Item response curves of Ueno2008 for two raters with different range restriction characteristics.

The range restriction characteristic is described only by Ueno2008. In the model, the parameter $\epsilon_{rk}$ represents the characteristic. When $\epsilon_{rk}$ and $\epsilon_{r(k-1)}$ are brought closer together, the probability of responding with category *k* decreases. Conversely, as the difference $\epsilon_{rk}-\epsilon_{r(k-1)}$ increases, the response probability for category *k* also increases. Figure 3 depicts the IRCs of the Ueno2008 for two raters with different $\epsilon_{rk}$ values. We used $\epsilon_{r1}=-2.5$, $\epsilon_{r2}=0.0$, $\epsilon_{r3}=1.0$, and $\epsilon_{r4}=3.0$ for the left panel. It has larger values of $\epsilon_{r2}-\epsilon_{r1}$ and $\epsilon_{r4}-\epsilon_{r3}$. The response probabilities for categories 2 and 4 are increased in the IRC. For the right panel, we set $\epsilon_{r1}=-3.0$, $\epsilon_{r2}=-2.0$, $\epsilon_{r3}=0.5$, and $\epsilon_{r4}=2.0$. The IRC shows that the response probability for category 3 is increased because $\epsilon_{r3}-\epsilon_{r2}$ has a larger value. The points presented above illustrate that the parameter $\epsilon_{rk}$ reflects the range restriction characteristic.

**Figure 4:**
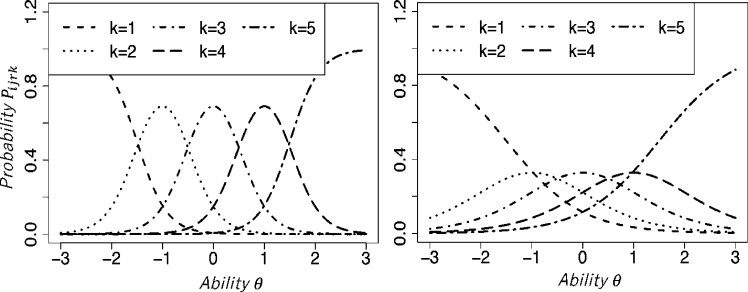
Item response curves of Uto2016 for two raters with different rating consistency.

Rater consistency is represented in Uto2016 and HRM by $\alpha_{r}$ and $1/\psi_{r}^{2}$. The lower the rater consistency parameter is, the smaller the differences in the response probabilities between the rating categories are. That fact reflects that a rater with a lower consistency parameter has a stronger tendency to assign different ratings to examinees with similar ability levels. Figure 4 presents IRCs of Uto2016 for two raters with different consistency levels. Here, the higher consistency value $\alpha_{r}=2.0$ is assigned to the left panel. The lower value $\alpha_{r}=0.8$ is assigned to the right panel. As a result, in the right IRC, the differences in the response probability among the categories are small.

The interpretation of task characteristics is similar to that of the item characteristic parameters explained in Subsection 3.4.

From the above, it is apparent that the previous models represent different types of rater and task characteristics.

### 5.2 Comparison of the numbers of parameters

The accuracy of parameter estimation generally decreases as the number of parameters per datum increases, as explained in Subsection 3.5. As the parameter estimation accuracy decreases, the accuracy of ability measurement generally declines (Uto and Ueno, 2016). Therefore, the number of parameters in a model is an important point for elucidating model features. For that reason, this subsection compares the numbers of parameters in the various models.

The last column of Table 1 shows the number of parameters in each model. The result shows that the MFRM has the fewest parameters. Therefore, the MFRM is expected to give the most accurate parameter estimation. However, as described earlier, the MFRM can represent only few rater and task characteristics. Therefore, if complex characteristics are assumed to occur in an assessment situation, then the MFRM might not fit the rating data.

In the other models, Uto2016 has the fewest parameters for numerous raters, such as for 2(R+1)>3I given I≥2 and K=5. Conversely, when the number of tasks is greater than the number of raters, specifically, 2(R+1)<3I, Ueno2008 has the fewest parameters. When the number of examinees is larger than the number of raters or items, HRM has the largest number of parameters because the number of ideal rating parameters $\xi_{ij}$ is increased.

### 5.3 Comparisons of parameter estimation accuracy

This subsection presents investigation of how the number of parameters affects the accuracy of parameter estimation and ability measurement. The number of parameters in each model is determined by the number of examinees, raters, and tasks, as explained before. Therefore, we evaluate the accuracy of each model with changing of their numbers. This experiment is conducted using simulation data to evaluate only the effects of the number of parameters, and to obtain data with various numbers of examinees, raters, and tasks. The procedures of this experiment are described below.

1. True parameters of MFRM, Patz1999, Uto2016, Ueno2008, and HRM were generated randomly for the following settings.
  (a) J=100,R=10,I=5,K=5
  (b) J=100,R=5,I=10,K=5
  (c) J=100,R=5,I=5,K=5
  (d) J=50,R=5,I=5,K=5
  (e) J=30,R=30,I=5,K=5
  Here, the model parameters were drawn from the distributions in equations (14), (15), (16), and (17).
  (14) $$\log\alpha_{i}∼N(0.1,0.4)$$
  (15) $$\log\alpha_{r},\log\psi_{r}∼N(0.0,0.5)$$
  (16) $$\beta_{i},\beta_{r},\beta_{ik},\varepsilon_{r},\rho_{ir},d_{ik},d_{k},b_{i},\sigma_{r},\theta_{j}∼N(0.0,1.0)$$
  (17) $$b_{ik},\epsilon_{rk}∼MN(\mu ,\Sigma )\left\{ \begin{array}{c} \mu =\{-1.50,-0.75,0.75,1.50\},\\ \Sigma =\left(\begin{array}{cccc} 0.25\; & 0.16\; & 0.16\; & 0.16\\ 0.16\; & 0.25\; & 0.16\; & 0.16\\ 0.16\; & 0.16\; & 0.25\; & 0.16\\ 0.16\; & 0.16\; & 0.16\; & 0.25 \end{array}\right) \end{array}\right.$$
2. Rating data ***U*** were sampled randomly from each model given the true parameters.
3. From the sampled data, the parameters of each model were estimated. This study used an expected a posteriori (EAP) estimation with the Markov Chain Monte Carlo (MCMC) algorithm (Fox, 2010; Patz and Junker, 1999; Uto and Ueno, 2016) because it is generally more robust for complex models than the other methods are (e.g., marginal maximum likelihood estimation or maximum a posteriori estimation) (Baker and Kim, 2004; Bishop, 2006; Fox, 2010). Here, the EAP estimates were calculated as the means of samples obtained from the 10,000 period to the 20,000 period at intervals of 100.
4. The root mean square error (RMSE) between the estimated parameters and true parameters was calculated. In our experiments, the accuracy of parameter estimation and ability measurement were evaluated using RMSE. Lower RMSE values indicate higher accuracy. This index has generally been used for evaluation of accuracy (e.g., Ilhan, 2016; Martin-Fernandez and Revuelta, 2017; Uto and Ueno, 2016; Wollack et al., 2002).
5. After repeating the procedures described above 10 times, the average and standard deviation of the RMSE values were calculated.

Table 2 presents the average and standard deviation (in parentheses) of RMSE over all raters and task characteristic parameters in each model. The results show that a lower number of parameters produces higher accuracy of parameter estimation. Specifically, MFRM having the fewest parameters achieved the highest accuracy. Also, HRM having the most parameters had the lowest accuracy among all settings. In addition, when the number of raters increased, Uto2016 having the second fewest parameters revealed the second highest accuracy. Conversely, when the number of tasks increased, Ueno2008 has the second fewest parameters and achieved the second highest accuracy.

**Table 2:** RMSE for rater and task parameters calculated in the simulation experiment.

	J=100	J=100	J=100	J=50	J=30
	R=10	R=5	R=5	R=5	R=30
	I=5	I=10	I=5	I=5	I=5
MFRM	.054 (.048)	.070 (.069)	.069 (.056)	.096 (.091)	.103 (.082)
Patz1999	.106 (.094)	.118 (.109)	.107 (.095)	.161 (.137)	.178 (.154)
Ueno2008	.108 (.089)	.073 (.074)	.119 (.102)	.161 (.130)	.189 (.189)
Uto2016	.088 (.091)	.078 (.081)	.105 (.091)	.130 (.110)	.127 (.114)
HRM	.252 (.283)	.335 (.493)	.477 (.467)	.349 (.331)	.223 (.252)

Furthermore, in all models except for HRM, the parameter estimation accuracy increases as the number of examinees increases. The accuracy of HRM did not increase because the number of parameters becomes large when examinees are numerous, as described in Subsection 5.2. Here, the accuracy of HRM increased as the number of raters increased because the increase of the number of parameters with the number of raters is low.

In addition, Table 3 presents the average and standard deviation (in parentheses) of RMSE for the ability. It shows that accuracy improves as the number of raters or tasks increases in all the models. In traditional IRT models, an increase of test items has a positive effect on improving the accuracy of ability measurement (e.g. Baker and Kim, 2004; van der Linden and Pashley, 2000). Our experimentally obtained result is consistent with this fact.

**Table 3:** RMSE for ability calculated in the simulation experiment.

	J=100	J=100	J=100	J=50	J=30
	R=10	R=5	R=5	R=5	R=30
	I=5	I=10	I=5	I=5	I=5
MFRM	.148 (.112)	.158 (.125)	.205 (.162)	.226 (.170)	.137 (.095)
Patz1999	.152 (.114)	.153 (.122)	.182 (.143)	.190 (.157)	.175 (.110)
Ueno2008	.166 (.130)	.150 (.116)	.211 (.161)	.214 (.151)	.151 (.115)
Uto2016	.159 (.129)	.155 (.117)	.177 (.125)	.193 (.147)	.145 (.107)
HRM	.371 (.299)	.302 (.239)	.379 (.290)	.385 (.295)	.403 (.316)

Furthermore, Table 3 shows that HRM presented the worst accuracy. The reason for this is the fact that the accuracy of parameter estimation in the model was extremely low. Comparison of the other models reveals that when the number of raters becomes large such as in the setting (e), MFRM and Uto2016 incorporating lower dimensional rater parameters presented higher ability measurement accuracy. In the other settings, all the models except for HRM exhibited comparable accuracy because their parameter estimation accuracy was high, although they were slightly different.

### 5.4 Model comparison for diverse characteristics of raters and tasks

The previous section demonstrated that the accuracy of parameter estimation and ability measurement depends on the number of parameters when the true model generating data is known. However, when the true model is unknown, the accuracy of ability measurement also depends on whether the model can precisely represent the rater and task characteristics appearing in an assessment process, as we discussed in Subsection 3.5. Consequently, this subsection presents evaluation of the effects of each rater and task characteristic for the accuracy. This experiment is also conducted through simulation to generate data with biases of specific rater and task characteristics.

In this experiment, rating data are first sampled from the MFRM, which is the simplest model. Then the data are transformed while reflecting each bias of rater and task characteristic listed in Table 4. Here, each rule is applied to randomly selected 60% raters or tasks, assuming more than half raters or tasks have the characteristics related to the rule. In each rule, 70% ratings in the data of each selected rater or task are transformed to biased data. When the number of categories K=5, the chance level that a rating matches an ideal rating is 20% even if ratings are provided randomly. In practice, a percentage of data greater than this is expected to be a valid rating. Consequently, in the rules, 30% data are not changed; 70% data are transformed.

**Table 4:** Transformation rules corresponding to assessment settings in which some rater and task characteristics are assumed to be present.

	Settings	Transformation procedure
(A)	Raters with low consistency exist	For 60% of raters *r*, ***U***_*r*_ is transformed to $U_{r}^{\prime }$ by changing 70% of the ratings to randomly selected rating categories.

(B)	Low discrimination tasks exist	For 60% of tasks *i*, ***U***_*i*_ is transformed to $U_{i}^{\prime }$ by changing 70% of the ratings to randomly selected rating categories.

(C)	Raters with strong range restriction exist	Two categories *k*^′^ and *k*^″^ (where *k*^′^ > *k*^″^) were first selected randomly. For 60% of raters *r*, ***U***_*r*_ is transformed to $U_{r}^{\prime }$ by changing 70% of the ratings to *k*^′^ if the rating is more than the average point of $U_{r}^{\prime }$, and changing it to *k*^″^ otherwise.

(D)	Difficulty to obtain each category differs among tasks	Two categories *k*^′^ and *k*^″^ (where *k*^′^ > *k*^″^) were first selected randomly. For 60% of tasks *i*, ***U***_*i*_ is transformed to $U_{i}^{\prime }$ by changing 70% of the ratings to *k*^′^ if the rating is more than the average point of $U_{i}^{\prime }$, and changing it to *k*^″^ otherwise.

(E)	Rater severity differs among tasks	We first selected *k*^‴^ ∈ {−*K* + 1,⋯,−1,1,⋯,*K* − 1} randomly. For 60% of task *i* and rater *r*, ***U***_*i*_ is transformed to $U_{ir}^{\prime }$ by changing 70% of rating $x_{ijr}^{\prime }\in U_{ir}$ to $x_{ijr}^{\prime }=x_{ijr}+k^{\prime \prime \prime }$ (where $x_{ijr}^{\prime }=1$ if $x_{ijr}^{\prime }<1$, and $x_{ijr}^{\prime }=K$ if $x_{ijr}^{\prime }>K$).

(F)	All the above characteristics exist	All the above transformation rules are applied simultaneously.

Using the data, the experiment compares the models based on the information criterion and the ability measurement accuracy. As described in Subsection 3.5, realizing accurate ability measurement can be facilitated by selection of an optimal model that can precisely represent bias factors using the fewest parameters. The information criterion generally selects a model with an appropriate tradeoff between goodness of fit to data and model complexity. Therefore, a model selected by the information criteria is expected to provide higher accuracy of ability measurement.

As information criteria, we use the Akaike Information Criterion (AIC, Akaike, 1974), the Widely Applicable Information Criterion (WAIC, Watanabe, 2010), the Bayesian Information Criterion (BIC, Schwarz, 1978), and the log Marginal Likelihood (ML). Of those, AIC and WAIC select a model that minimizes the generalization error, which is regarded as the prediction error for future data. ML and BIC realize consistent model selection, which means that the probability of selecting the true model goes to 1.0 as the data size approaches infinity. Both AIC and BIC have been used widely for IRT model selection because they are easily calculated (Fox, 2010; Nering and Ostini, 2010; Reise and Revicki, 2014; Uto and Ueno, 2016; van der Linden, 2016b). Both WAIC and ML have recently become popular with the widespread use of MCMC (e.g., Almond, 2014; Eric, 2008; Luo and Al-Harbi, 2017; Uto et al., 2017b; Vehtari et al., 2017) because they are calculable using MCMC samples (Newton and Raftery, 1994; Watanabe, 2010). Also, WAIC and ML are expected to provide better results than AIC and BIC do because WAIC is a generalization of AIC, and because BIC is an asymptotic approximation of ML. In those criteria, the model which maximizes the score is regarded as the optimal model.

The procedures of this experiment were the following.

1. For J=100, R=5, I=5, and K=5, the true parameters of MFRM were selected randomly following the distributions in equation (16).
2. Given the true parameters, rating data ***U*** were sampled from MFRM.
3. Data ***U*** were transformed to $U^{\prime }$ by applying a rule in Table 4. In Table 4, $U_{r}=\{x_{ijr}|r\;\text{fixed}\}\subset U$, $U_{i}=\{x_{ijr}|i\;\text{fixed}\}\subset U$, $U_{ir}=\{x_{ijr}|i,r\;\text{fixed}\}\subset U$.
4. From each of the processed datasets $U^{\prime }$, we estimated the parameters of MFRM, Patz1999, Ueno2008, Uto2016, and HRM by MCMC.
5. The models were ranked based on results of model selections using information criteria.
6. RMSEs between the true ability and the ability estimates obtained from each model were calculated.
7. After repeating the procedure described above 10 times, we calculated the average and standard deviation of the ranks and RMSEs.

Table 5 presents the average and standard deviation (in parentheses) of the estimated ranks and the RMSEs. In the table, bold typeface text represents the lowest rank and RMSE. Results show that the model performance depends strongly on whether the model can represent the rater and task characteristics appearing in an assessment process, in addition to the number of model parameters. Specifically, the following findings were obtained from the results.

• For data (A), in which raters with lower consistency exist, Uto2016 was selected as the optimal model by all information criteria. Furthermore, the model presented the highest accuracy of ability measurement. The result indicates that the model can appropriately represent the characteristics of raters with low consistency, and that it can estimate the ability considering its effects. Results show that HRM did not present high performance, although it also has the rater consistency parameter. The reason is that the parameter estimation accuracy is extremely low, as explained in the previous section.
• For data (B), in which tasks with lower discrimination exist, Patz1999 had the highest information criteria and ability measurement accuracy. Furthermore, Uto2016 and Ueno2008 had similar ability measurement accuracy. The results suggest that the incorporation of task discrimination parameters is necessary to improve the accuracy when varying task discrimination is assumed.
• For data (C), Ueno2008 presented the highest performance. The result indicates that the use of Ueno2008 is recommended when raters with strong range restriction exist because only this model can represent the relevant characteristic.
• For data (D), in which the difficulties in obtaining each category differ among tasks, Uto2016 and Patz1999 presented equally high performances because these models can represent this characteristic. Although HRM can also represent this characteristic, it showed poor performance because the parameter estimation accuracy was extremely low, as discussed before.
• For data (E), in which the severity of raters changed among the tasks, Patz1999 presented the best performance because only this model can represent the relevant characteristic. According to the result, Patz1999 is expected to perform well when interactions between raters and tasks are assumed to exist.
• For data (F), for which all the above rater and task characteristics exist, all models presented extremely low ability measurement accuracies because no model can incorporate all those rater and task characteristics simultaneously. That result suggests that another model with a higher dimensional rater and task parameters will be required for such circumstances.

**Table 5:** Performance of models in various assessment settings.

Setting	Model	AIC	WAIC	BIC	ML	RMSE(*θ*)
(A)	MFRM	4.50(.45)	4.20(.36)	3.90(.09)	4.90(.09)	.478(.048)
	Patz1999	2.10(.09)	2.10(.09)	2.10(.09)	2.10(.09)	.404(.042)
	Ueno2008	2.89(.10)	2.89(.10)	2.89(.10)	2.89(.10)	.394(.036)
	Uto2016	**1.00**(.00)	**1.00**(.00)	**1.00**(.00)	**1.00**(.00)	**.295**(.028)
	HRM	4.30(.21)	4.60(.24)	4.90(.09)	3.90(.09)	.478(.068)

(B)	MFRM	4.80(.16)	4.70(.21)	3.90(.09)	4.90(.09)	.548(.058)
	Patz1999	**1.00**(.00)	**1.00**(.00)	**1.00**(.00)	**1.00**(.00)	**.353**(.025)
	Ueno2008	3.00(.00)	3.00(.00)	3.00(.00)	3.00(.00)	.392(.047)
	Uto2016	2.00(.00)	2.00(.00)	2.00(.00)	2.00(.00)	.373(.026)
	HRM	4.00(.20)	4.10(.29)	4.90(.09)	3.90(.09)	.635(.115)

(C)	MFRM	4.00(.00)	4.00(.00)	4.00(.00)	4.30(.21)	.318(.069)
	Patz1999	2.60(.24)	2.60(.24)	2.60(.24)	2.60(.24)	.258(.035)
	Ueno2008	**1.00**(.00)	**1.00**(.00)	**1.00**(.00)	**1.00**(.00)	**.236**(.031)
	Uto2016	2.40(.24)	2.40(.24)	2.40(.24)	2.40(.24)	.255(.035)
	HRM	5.00(.00)	5.00(.00)	5.00(.00)	4.70(.21)	.385(.047)

(D)	MFRM	4.00(.00)	4.00(.00)	4.00(.00)	4.40(.24)	.318(.057)
	Patz1999	**1.50**(.25)	**1.50**(.25)	1.60(.24)	**1.50**(.25)	.259(.026)
	Ueno2008	3.00(.00)	3.00(.00)	3.00(.00)	3.00(.00)	.286(.028)
	Uto2016	**1.50**(.25)	**1.50**(.25)	**1.40**(.24)	**1.50**(.25)	**.252**(.027)
	HRM	5.00(.00)	5.00(.00)	5.00(.00)	4.60(.24)	.408(.054)

(E)	MFRM	4.40(.24)	4.60(.24)	4.00(.20)	4.90(.09)	.419(.065)
	Patz1999	**1.00**(.00)	**1.00**(.00)	**1.00**(.00)	**1.00**(.00)	**.285**(.029)
	Ueno2008	2.89(.10)	2.89(.10)	2.89(.10)	2.89(.10)	.343(.055)
	Uto2016	2.10(.09)	2.10(.09)	2.10(.09)	2.10(.09)	.350(.050)
	HRM	4.40(.44)	4.20(.36)	4.80(.16)	3.90(.09)	.711(.162)

(F)	MFRM	4.90(.09)	4.90(.09)	4.80(.16)	4.90(.09)	.735(.051)
	Patz1999	**1.00**(.00)	**1.00**(.00)	**1.00**(.00)	**1.00**(.00)	**.671**(.069)
	Ueno2008	3.00(.00)	3.00(.00)	3.00(.00)	3.00(.00)	.708(.066)
	Uto2016	2.00(.00)	2.00(.00)	2.00(.00)	2.00(.00)	.691(.102)
	HRM	3.90(.09)	3.90(.09)	4.00(.20)	3.90(.09)	.876(.062)

### 5.5 Actual data experiments

Summarizing the discussion in the simulation experiments, we conclude that the IRT models performance depends strongly on 1) the number of parameters and 2) the assumed rater and task characteristics. This section validates the conclusions through model applications to two actual datasets.

#### 5.5.1 Actual data

This study uses the following two rating datasets obtained from subject experiments.

1. *Report assessment data*: The data consist of ratings provided by five raters to reports that were submitted by 30 university students for five tasks. Here, the tasks were provided during an e-learning course. The raters were course tutors.
2. *Peer assessment data*: The data consist of ratings assigned to essays written by 30 university students for four writing tasks. Here, all the students assessed each other.

In both assessments, the ratings were conducted using a rubric consisting of five rating categories.

#### 5.5.2 Descriptive statistics

To analyze what types of rater and task characteristics can be assumed in each dataset, this subsection presents an analysis based on descriptive statistics.

Rater severity is usually evaluated by the averaged ratings of each rater (Saal et al., 1980). A rater can be regarded as severe if the averaged rating is less than the midpoint of the rating scale. Task difficulty is also evaluated by the averaged rating assigned for each task. Range restriction can be assessed from the rate of appearance of each category. Raters might have range restriction characteristics if they have overused a few categories. Rater consistency is often estimated as the point-biserial correlation between the ratings given by a rater and the total ratings given by all the raters excluding the rater (DeMars, 2010). In classical test theory, the correlation is known as item–rest correlation (I-R correlation) (Bechger et al., 2003). A higher I-R correlation signifies that the rater is consistently giving ratings that are correlated well with the true ability level. Similarly, the I-R correlation between the ratings on a task and the total ratings on all tasks excluding the task is used as an index of the task discrimination. It is noteworthy that the indices presented here are inappropriate for categorical data. However, they have been used widely for analyzing performance assessment data because of their simplicity and ease of calculation.

Table 6 and Table 7 present averages of the ratings, I-R correlation, and the appearance rate of each category for all raters and tasks for the two datasets. In the tables, the *Avg.* column presents the average rating of each rater or task, the *I-R Cor* column shows the I-R correlation, and columns for k=1,⋯,5 in *Appearance rate for each category* column show the rates of the respective categories. Here, for the analysis of whether the rater severity differs among the tasks, the average scores of raters for each task are also presented in the *Average scores of raters for each task* column.

**Table 6:** Descriptive statistics for the report assessment data.

	Avg.	I-R Cor	Appearance rate for each category					Average scores of raters for each task				
			1	2	3	4	5	1	2	3	4	5
Rater 1	1.820	0.781	9.8	32.9	30.1	19.6	7.7	1.852	1.933	1.704	1.483	2.133
Rater 2	1.962	0.785	6.3	30.8	33.6	19.6	9.8	1.741	2.033	1.778	2.103	2.100
Rater 3	2.268	0.651	2.0	10.1	51.5	34.3	2.0	2.375	2.167	2.321	2.167	2.667
Rater 4	2.507	0.652	0.0	3.5	49.3	39.6	7.6	2.296	2.467	2.464	2.586	2.733
Rater 5	2.705	0.739	0.7	7.4	35.8	31.8	24.3	2.533	2.633	2.897	2.759	2.767

Task 1	2.128	0.533	7.6	18.5	38.7	23.5	11.8					
Task 2	2.247	0.750	5.3	13.3	44.0	26.0	11.3					
Task 3	2.180	0.414	2.2	20.9	38.8	26.6	11.5					
Task 4	2.160	0.651	4.1	19.2	36.3	31.5	8.9					
Task 5	2.428	0.669	0.0	14.6	38.2	35.8	11.4

**Table 7:** Descriptive statistics for the peer assessment data.

	Avg.	I-R Cor	Appearance rate for each category					Average scores of raters for each task			
			1	2	3	4	5	1	2	3	4
Rater 1	2.392	0.590	2.5	19.2	28.3	36.7	13.3	1.933	2.400	2.533	2.700
Rater 2	2.325	0.673	10.8	13.3	24.2	35.8	15.8	1.900	2.433	2.467	2.500
Rater 3	1.842	0.631	8.3	27.5	40.8	18.3	5.0	1.800	1.800	1.900	1.867
Rater 4	2.367	0.491	0.8	15.8	32.5	47.5	3.3	2.000	2.433	2.533	2.500
Rater 5	2.492	0.408	0.0	13.3	38.3	34.2	14.2	2.300	2.500	2.567	2.600
Rater 6	2.333	0.406	0.8	20.0	33.3	36.7	9.2	2.367	2.400	2.133	2.433
Rater 7	1.258	0.500	31.7	27.5	29.2	6.7	5.0	1.433	0.900	1.333	1.367
Rater 8	1.992	0.568	0.8	16.7	65.8	15.8	0.8	1.967	1.867	1.900	2.233
Rater 9	1.450	0.451	7.5	50.8	30.8	10.8	0.0	1.733	1.533	1.000	1.533
Rater 10	2.625	0.733	6.7	13.3	21.7	27.5	30.8	2.400	2.567	2.700	2.833
Rater 11	2.517	0.525	0.0	9.2	40.0	40.8	10.0	2.800	2.367	2.300	2.600
Rater 12	2.392	0.470	0.0	12.5	42.5	38.3	6.7	2.300	2.333	2.367	2.567
Rater 13	1.525	0.522	15.0	38.3	30.8	10.8	5.0	1.833	1.567	1.300	1.400
Rater 14	1.908	0.380	3.3	34.2	35.8	21.7	5.0	1.767	2.133	1.733	2.000
Rater 15	2.383	0.546	0.0	7.5	50.8	37.5	4.2	2.200	2.300	2.467	2.567
Rater 16	2.575	0.533	4.2	1.7	29.2	62.5	2.5	2.200	2.633	2.767	2.700
Rater 17	2.683	0.493	0.0	5.0	35.8	45.0	14.2	2.467	2.900	2.467	2.900
Rater 18	2.108	0.626	1.7	21.7	44.2	29.2	3.3	2.233	2.000	2.067	2.133
Rater 19	1.683	0.461	0.0	32.5	66.7	0.8	0.0	1.767	1.567	1.733	1.667
Rater 20	1.717	0.540	5.8	33.3	44.2	16.7	0.0	1.633	1.533	1.567	2.133
Rater 21	2.225	0.676	6.7	24.2	28.3	21.7	19.2	2.067	2.100	2.267	2.467
Rater 22	1.883	0.538	0.8	29.2	51.7	17.5	0.8	1.700	1.800	1.900	2.133
Rater 23	2.150	0.197	0.8	7.5	68.3	22.5	0.8	2.067	2.233	2.033	2.267
Rater 24	2.008	0.247	7.5	25.0	36.7	20.8	10.0	1.867	1.867	2.167	2.133
Rater 25	2.600	0.650	6.7	15.8	20.8	24.2	32.5	2.067	2.700	2.533	3.100
Rater 26	1.533	0.481	20.8	34.2	22.5	15.8	6.7	2.233	1.267	1.433	1.200
Rater 27	2.592	0.663	4.2	15.0	16.7	45.8	18.3	2.500	2.667	2.500	2.700
Rater 28	2.875	0.334	0.8	3.3	17.5	64.2	14.2	2.900	2.867	2.767	2.967
Rater 29	2.142	0.644	2.5	21.7	41.7	27.5	6.7	2.100	2.033	2.000	2.433
Rater 30	2.500	0.706	1.7	25.0	15.8	36.7	20.8	1.933	2.567	2.833	2.667

Task 1	2.082	0.474	6.8	23.2	34.2	26.6	9.2				
Task 2	2.142	0.538	5.8	21.0	35.9	27.9	9.4				
Task 3	2.142	0.535	4.6	20.9	37.9	29.1	7.6				
Task 4	2.310	0.587	3.2	16.8	36.7	32.4	10.9				

Table 6 and Table 7 show that the average ratings varied across the raters for each data group, which reflects that the raters have different severity characteristics.

Furthermore, we can confirm from the tables that some raters might have a strong range restriction for each data group. The distribution of the appearance rate for the categories in a rater generally becomes unimodal with a peak at a central category because the abilities of examinees generally follow a normal distribution. Moreover, it is desirable that a rater use all categories to discriminate the ability of an examinee more clearly. Therefore, *Rater 1* and *2* in *report assessment data*; those of *Rater 14* and *21* in *peer assessment data* can be regarded as desirable raters, for example. From comparison to them, we can confirm that the distributions of some raters are skewed. For example, about 85%∼90% of ratings given by *Rater 3* and *4* in *report assessment data* are concentrated in categories 3 and 4. Similarly, those given by *Rater 9*, and *19* in *peer assessment data* were concentrated in categories 2 and 3. This analysis suggests that these raters have stronger range restriction characteristics. Although we showed the examples of overusing two adjacent categories from the tables, various patterns of range restriction can practically occur, such as overusing the extreme categories and only a single specific category (e.g. Eckes, 2015; Kassim, 2011; Myford and Wolfe, 2003).

Furthermore, according to Table 6 and Table 7, the I-R correlations for raters were not so different in the *report assessment data*, but they were different in the *peer assessment data*. This result suggests that the variety of rater consistency might be large in the *peer assessment data*.

Moreover, Table 6 and Table 7 show that the rater severity was not so different among the tasks in both data groups. The ratings for *Task 5* in the *report assessment data* and those for *Task 4* in the *peer assessment data* were slightly higher than for the other tasks. However, the reason is that the task difficulty was lower than the other tasks, as we can confirm from the *Avg.* column. Similarly, the reason why the ratings for *Task 1* in the *peer assessment data* were low is that the difficulty was high.

In addition, Table 6 shows that the I-R correlations for tasks varied in the *report assessment data*. In these data, therefore, the tasks might have different discrimination powers. Moreover, comparison of Table 6 with Table 7 reveals that the variety of I-R correlations for tasks in the *peer assessment data* was smaller than that in the *report assessment data*, which suggests that the impact of using the task discrimination parameters will be high for the *report assessment data*. We can confirm that the other task characteristics did not vary greatly.

From the previous discussion, we can predict that Ueno2008 will be suitable for the *report assessment data* because a large variety of range restriction and task discrimination were confirmed but the differences of consistency might not be large. For the *peer assessment data*, Uto2016 is expected to achieve high performance because raters with different consistency were detected and because the model incorporates the second fewest parameters in these settings. Although HRM can also consider the rater consistency, it will not perform better because the accuracy of ability measurement is extremely low, as explained in the previous section.

#### 5.5.3 Comparisons using information criteria

This subsection compares the IRT models based on the information criteria introduced in Subsection 5.4. The experimental procedures were the following.

1. For each dataset, the parameters used for MFRM, Patz1999, Ueno2008, Uto2016, and HRM were estimated using the MCMC algorithm.
2. Using the estimation results, AIC, WAIC, BIC, and ML were calculated.

Table 8 presents the results. In the table, bold typeface text denotes maximum scores.

**Table 8:** Information criterion values calculated from actual data.

Data		AIC	WAIC	BIC	ML
Report assessment data	MFRM	−809.186	−803.968	−838.611	−786.042
	Patz1999	−826.134	−815.524	−875.176	−787.831
	Ueno2008	**−779.449**	**−779.449**	**−831.401**	**−756.119**
	Uto2016	−807.605	−797.879	−851.743	−771.613
	HRM	−1050.488	−1445.299	−1197.613	−868.446

Peer assessment data	MFRM	−4650.06	−4646.46	−4696.3	−4615.25
	Patz1999	−4662.97	−4646.08	−4776.47	−4575.41
	Ueno2008	−4541.02	−4504.17	−4651.02	−4445.21
	Uto2016	**−4442.92**	**−4434.82**	**−4518.58**	**−4385.57**
	HRM	−4683.719	−7035.085	−4842.054	−4498.075

Table 8 shows that Ueno2008 was selected as the optimal model based on all information criteria for the *report assessment data*. From the discussion in Subsection 5.5.2, this result derives from the rater consistency uniformity, the large variety of the range restriction among raters, and that of the discrimination among tasks.

For *peer assessment data*, Uto2016 was selected as the optimal model based on the following reasons: 1) Consistency differs among raters. 2) Higher accuracy of parameter estimation can be realized because the model has the second fewest parameters in the models when the number of raters increases as in this dataset.

#### 5.5.4 Comparisons of ability measurement accuracy

This subsection presents a comparison of the ability measurement accuracy using the actual datasets.

In the simulation experiments, we evaluated the accuracy using the error between the true ability and the estimated ability values. However, in actual data experiments, the true ability is unknown. Therefore, we evaluate it based on the error between the ability estimated using complete data and that estimated using a subset of the data. The subset of the data is created by changing some rating data to missing data. Here, we create the missing data assuming the judge pair design (Eckes, 2015; Ilhan, 2016), which assigns only two raters to each outcome. A model that can measure the ability with little error when using fewer ratings is regarded as an accurate model (Uto and Ueno, 2016).

For accuracy evaluation according to this idea, the following experiment was conducted.

1. For each dataset, the parameters of MFRM, Patz1999, Ueno2008, Uto2016, and HRM were estimated using the MCMC algorithm.
2. Assuming the judge pair design, two raters were assigned to each outcome. Then, the ratings given by the raters who were not assigned to each outcome were changed to missing data.
3. Using the missing data, ability was estimated given the rater and task parameters estimated in procedure 1.
4. We calculated the RMSE, mean absolute error (MAE), and standard deviation (SD) of the absolute error between the ability estimated using the complete data and that estimated with the missing data.

Table 9 shows the results. In the table, bold typeface text represents the lowest RMSE and MAE values. From the results presented in Table 9, one can confirm that the models with higher values of the information criterion tend to provide lower RMSEs and MAEs. Concretely, Ueno2008 had the highest accuracy for the *report assessment data*, and Uto2016 had the highest accuracy for the *peer assessment data*. The tendency is consistent with those of the simulation experiments described in Subsection 5.4.

**Table 9:** Ability measurement error calculated from actual data.

	Report assessment data			Peer assessment data		
	RMSE	MAE	SD	RMSE	MAE	SD
MFRM	0.337	0.254	0.221	0.334	0.258	0.212
Patz1999	0.382	0.319	0.211	0.360	0.285	0.219
Ueno2008	**0.238**	**0.154**	0.181	0.316	0.229	0.217
Uto2016	0.253	0.187	0.171	**0.233**	**0.181**	0.146
HRM	0.422	0.321	0.274	0.453	0.330	0.311

Therefore, we confirmed that the model which appropriately reflects the rater and task characteristics assumed in the data and which has as few parameters as possible can achieve higher accuracy for ability measurement.

## 6 Discussion

The discussions and experimentally obtained results in this study show that the accuracy of ability measurement using IRT models depends on the following two points: 1) The characteristics of raters and tasks which are assumed to be present in the assessment process are modeled appropriately. 2) The parameters are as few as possible because the accuracy of parameter estimation and ability measurement generally decreases as the number of parameters increases. Based on those points, this subsection presents a summary of the model features.

The main feature of the MFRM is that it is defined by the fewest parameters of all models. Consequently, the MFRM can estimate model parameters from a small dataset more accurately than the other models can. Therefore, the model will be suitable when a large amount of rating data cannot be obtained. However, the MFRM can represent only a few rater and task characteristics. Therefore, if complex characteristics are assumed to occur in an assessment situation, then the MFRM will not perform well.

A unique feature of Patz1999 is the incorporation of a different rater severity for each task. When the severity of raters is likely to change between tasks, the model will provide better performance. However, when the raters or tasks become numerous, the model performance will decline because the number of parameters increases rapidly.

Ueno2008 has the unique feature that it can represent the range restriction characteristics of raters. Therefore, the model will provide better performance when raters with a strong range restriction are likely to exist, as shown in the case of the simulation and actual data experiments. In addition, the model has another feature: the parameters are the second fewest when the tasks are most numerous. Therefore, the model is suitable when differences in range restriction among raters are likely and the number of tasks is large.

Uto2015 has two features: it incorporates a rater consistency parameter; and the parameters are the second fewest when the number of raters is large. Therefore, the model is suitable when the rater consistency is likely to vary and the raters are numerous, as was the case for the peer assessment data in the actual data experiment.

HRM is developed based on a different modeling method. The model includes the assumption that each outcome of an examinee for a task has an ideal score. Therefore, the model would be useful for estimating those scores directly. However, the parameter estimation accuracy declines as the examinees become more numerous because the number of parameters in the model increases considerably. This feature is undesirable because the examinees are generally numerous in actual performance assessments. Therefore, the benefits of using HRM might be constrained in normal assessment situations.

## 7 Conclusion

This article described a comparison of IRT models that incorporate rater and task characteristic parameters. First, we examined representative rater and task characteristics that might affect the ability measurement accuracy. Then, we introduced existing IRT models incorporating rater and task characteristic parameters. We also summarized and explained the rater and task characteristics assumed for each model. Through simulation experiments, we next demonstrated the relations between the number of parameters, the accuracy of parameter estimation, and ability measurement. Additionally, we evaluated the performance of each model when some specific characteristics of tasks and raters were assumed for assessment processes. Finally, we also compared the models using two sets of actual performance assessment data. Although the experimentally obtained results were only examples, we were able to confirm the features and benefits of each model from the data.

Actually, preparing a sufficient number and quality of tasks and raters is the most effective means of improving the accuracy of ability measurement (Eckes, 2015; Myford and Wolfe, 2003). However, ideal assessments might often be infeasible because of time and economic constraints. For such cases, the use of IRT models is a convenient alternative.

As explained in Subsection 3.5, the IRT models introduced in this study assume unidimensionality. However, in practical assessment situations, the existence of multidimensional ability might be assumed. For such cases, multidimensional IRT models incorporating rater characteristic parameters are expected to present better performance. Developing such models is left as a subject for future work.

Moreover, the models are useful not only for estimating examinee ability but also for various other purposes such as the evaluation of raters, tasks, and rubric, or recommending optimal raters and tasks for each examinee. Some applications of IRT models for such purposes have recently been proposed (Nguyen et al., 2015; Uto et al., 2017a). In addition, the IRT models might be applicable to general rating data, such as item ratings in online shops and worker evaluation data in crowd sourcing system. We hope that, by providing this analysis, we support the development and use of more diverse applications.

## Declarations

### Author contribution statement

Masaki Uto: Conceived and designed the experiments; Performed the experiments; Analyzed and interpreted the data; Contributed reagents, materials, analysis tools or data; Wrote the paper.

Maomi Ueno: Conceived and designed the experiments; Analyzed and interpreted the data.

### Funding statement

This work was supported by JSPS KAKENHI Grant Numbers 17H04726.

### Competing interest statement

The authors declare no conflict of interest.

### Additional information

Data associated with this study has been deposited at Mendeley data under the accession numbers https://doi.org/10.17632/23wfdr9r5k.1 (Peer Assessment Data) and https://doi.org/10.17632/tv47gjd6pm.1 (Report Assessment Data).

## References

[br0010] Akaike H. (1974). A new look at the statistical model identification. IEEE Trans. Autom. Control.

[br0020] Almond R.G. (2014). A comparison of two MCMC algorithms for hierarchical mixture models. Proceedings of the Eleventh Uncertainty in Artificial Intelligence Conference on Bayesian Modeling Applications Workshop.

[br0030] Andrich D. (1978). A rating formulation for ordered response categories. Psychometrika.

[br0040] Baker F., Kim S.H. (2004). Item Response Theory: Parameter Estimation Techniques.

[br0050] Bechger T.M., Maris G., Verstralen H.H., Béguin A.A. (2003). Using classical test theory in combination with item response theory. Appl. Psychol. Meas..

[br0060] Bernardin H.J., Thomason S., Buckley M.R., Kane J.S. (2016). Rater rating-level bias and accuracy in performance appraisals: the impact of rater personality, performance management competence, and rater accountability. Hum. Resour. Manag..

[br0070] Bishop C.M. (2006). Pattern Recognition and Machine Learning.

[br0080] Carlson J.E., von Davier M. (2013). Item Response Theory.

[br0090] de Ayala R.J. (2009). The Theory and Practice of Item Response Theory.

[br0100] DeCarlo L.T. (2005). A model of rater behavior in essay grading based on signal detection theory. J. Educ. Meas..

[br0110] DeCarlo L.T., Kim Y.K., Johnson M.S. (2011). A hierarchical rater model for constructed responses, with a signal detection rater model. J. Educ. Meas..

[br0120] DeMars C. (2010). Item Response Theory.

[br0130] Eckes T. (2005). Examining rater effects in TestDaF writing and speaking performance assessments: a many-facet Rasch analysis. Lang. Assess. Q..

[br0140] Eckes T. (2015). Introduction to Many-Facet Rasch Measurement: Analyzing and Evaluating Rater-Mediated Assessments.

[br0150] Eric J.W. (2008). A review and comparison of four commonly used Bayesian and maximum likelihood model selection tools. Ecol. Model..

[br0160] Esfandiari R., Farrokhi F., Dalili M.V. (2013). Applying the many-facet Rasch model to detect centrality in self-assessment, peer-assessment and teacher assessment. World Appl. Sci. J..

[br0170] Fox J.-P. (2010). Bayesian Item Response Modeling: Theory and Applications.

[br0180] Goldin I.M. (2012). Accounting for peer reviewer bias with Bayesian models. Proc. the Workshop on Intelligent Support for Learning Groups at the 11th International Conference on Intelligent Tutoring Systems.

[br0190] Ilhan M. (2016). A comparison of the results of many-facet Rasch analyses based on crossed and judge pair designs. Educ. Sci.: Theory Pract..

[br0200] Information Technology Promotion Agency (2017). Information technology engineers examination registered information security specialist examination (ver 3.0). https://www.ipa.go.jp/files/000009648.pdf.

[br0210] Kassim N.L.A. (2011). Judging behaviour and rater errors: an application of the many-facet Rasch model. GEMA Online J. Lang. Stud..

[br0220] Linacre J. (1989). Many-Faceted Rasch Measurement.

[br0230] Lord F. (1980). Applications of Item Response Theory to Practical Testing Problems.

[br0240] Lu Y., Wang X. (2006). A Hierarchical Bayesian Framework for Item Response Theory Models with Applications in Ideal Point Estimation.

[br0250] Luo Y., Al-Harbi K. (2017). Performances of LOO and WAIC as IRT model selection methods. Psychol. Test Assess. Model..

[br0260] Martin-Fernandez M., Revuelta J. (2017). Bayesian estimation of multidimensional item response models. A comparison of analytic and simulation algorithms. Int. J. Methodol. Exp. Psychol..

[br0270] Masters G. (1982). A Rasch model for partial credit scoring. Psychometrika.

[br0280] Muraki E. (1992). A generalized partial credit model: application of an EM algorithm. Appl. Psychol. Meas..

[br0290] Muraki E., van der Linden W.J., Hambleton R.K. (1997). A generalized partial credit model. Handbook of Modern Item Response Theory.

[br0300] Muraki E., Hombo C., Lee Y. (2000). Equating and linking of performance assessments. Appl. Psychol. Meas..

[br0310] Myford C.M., Wolfe E.W. (2003). Detecting and measuring rater effects using many-facet Rasch measurement: part I. J. Appl. Meas..

[br0320] Myford C.M., Wolfe E.W. (2004). Detecting and measuring rater effects using many-facet Rasch measurement: part II. J. Appl. Meas..

[br0330] Nering M.L., Ostini R. (2010). Handbook of Polytomous Item Response Theory Models.

[br0340] Newton M., Raftery A. (1994). Approximate Bayesian inference by the weighted likelihood bootstrap. J. R. Stat. Soc., Ser. B, Methodol..

[br0350] Nguyen T., Uto M., Abe Y., Ueno M. (2015). Reliable peer assessment for team project based learning using item response theory. Proc. International Conference on Computers in Education.

[br0360] Palm T. (2008). Performance assessment and authentic assessment: a conceptual analysis of the literature. Pract. Assess., Res. Eval..

[br0370] Patz R.J., Junker B. (1999). Applications and extensions of MCMC in IRT: multiple item types, missing data, and rated responses. J. Educ. Behav. Stat..

[br0380] Patz R.J., Junker B.W., Johnson M.S., Mariano L.T. (1999). The hierarchical rater model for rated test items and its application to large-scale educational assessment data. J. Educ. Behav. Stat..

[br0390] Peterson W., Birdsall T., Fox W. (1954). The theory of signal detectability. Trans. IRE Prof. Group Inf. Theory.

[br0400] Piech C., Huang J., Chen Z., Do C., Ng A., Koller D. (2013). Tuned models of peer assessment in MOOCs. Proc. of Sixth International Conference of MIT's Learning International Networks Consortium.

[br0410] Rahman A.A., Ahmad J., Yasin R.M., Hanafi N.M. (2017). Investigating central tendency in competency assessment of design electronic circuit: analysis using many facet Rasch measurement (MFRM). Int. J. Inf. Educ. Technol..

[br0420] Reise S.P., Revicki D.A. (2014). Handbook of Item Response Theory Modeling: Applications to Typical Performance Assessment.

[br0430] Saal F., Downey R., Lahey M. (1980). Rating the ratings: assessing the psychometric quality of rating data. Psychol. Bull..

[br0440] Samejima F. (1969). Estimation of latent ability using a response pattern of graded scores. Psychom. Monogr..

[br0450] San Martín E., González J., Tuerlinckx F. (2015). On the unidentifiability of the fixed-effects 3PL model. Psychometrika.

[br0460] Schwarz G. (1978). Estimating the dimensions of a model. Ann. Stat..

[br0470] Shah N.B., Bradley J., Balakrishnan S., Parekh A., Ramchandran K., Wainwright M.J. (2014). Some scaling laws for MOOC assessments. ACM KDD Workshop on Data Mining for Educational Assessment and Feedback.

[br0480] Suen H. (2014). Peer assessment for massive open online courses (MOOCs). Int. Rev. Res. Open Distrib. Learn..

[br0490] Sung H.J., Kang T. (2006). Choosing a polytomous IRT model using Bayesian model selection methods. National Council on Measurement in Education Annual Meeting.

[br0500] Ueno M., Okamoto T. (2008). Item response theory for peer assessment. Proc. IEEE International Conference on Advanced Learning Technologies.

[br0510] Uto M., Duc Thien N., Ueno M. (2017). Group optimization to maximize peer assessment accuracy using item response theory. Proc. International Conference on Artificial Intelligence in Education.

[br0520] Uto M., Louvigné S., Kato Y., Ishii T., Miyazawa Y. (2017). Diverse reports recommendation system based on latent Dirichlet allocation. Behaviormetrika.

[br0530] Uto M., Ueno M. (2016). Item response theory for peer assessment. IEEE Trans. Learn. Technol..

[br0540] van der Linden W.J. (2016). Handbook of Item Response Theory, Volume One: Models.

[br0550] van der Linden W.J. (2016). Handbook of Item Response Theory, Volume Two: Statistical Tools.

[br0560] van der Linden W.J., Pashley P.J., van der Linden W.J., Glas G.A. (2000). Item selection and ability estimation in adaptive testing. Computerized Adaptive Testing: Theory and Practice.

[br0570] Vehtari A., Gelman A., Gabry J. (2017). Practical Bayesian model evaluation using leave-one-out cross-validation and WAIC. Stat. Comput..

[br0580] Waller M.I. (1981). A procedure for comparing logistic latent trait models. J. Educ. Meas..

[br0590] Watanabe S. (2010). Asymptotic equivalence of Bayes cross validation and widely applicable information criterion in singular learning theory. J. Mach. Learn. Res..

[br0600] Wollack J.A., Bolt D.M., Cohen A.S., Lee Y.-S. (2002). Recovery of item parameters in the nominal response model: a comparison of marginal maximum likelihood estimation and Markov chain Monte Carlo estimation. Appl. Psychol. Meas..

[br0610] Wren G.D. (2009). Performance Assessment: A Key Component of a Balanced Assessment System.

